# Hitting and missing targets by ambulance services for emergency calls: effects of different systems of performance measurement within the UK

**DOI:** 10.1111/j.1467-985X.2008.00557.x

**Published:** 2009-01

**Authors:** Gwyn Bevan, Richard Hamblin

**Affiliations:** London School of Economics and Political ScienceUK; Healthcare CommissionLondon, UK

**Keywords:** Ambulance response times, Performance measurement, Star ratings, Targets

## Abstract

Following devolution, differences developed between UK countries in systems of measuring performance against a common target that ambulance services ought to respond to 75% of calls for what may be immediately life threatening emergencies (category A calls) within 8 minutes. Only in England was this target integral to a ranking system of ‘star rating’, which inflicted reputational damage on services that failed to hit targets, and only in England has this target been met. In other countries, the target has been missed by such large margins that services would have been publicly reported as failing, if they had been covered by the English system of star ratings. The paper argues that this case-study adds to evidence from comparisons of different systems of hospital performance measurement that, to have an effect, these systems need to be designed to inflict reputational damage on those that have performed poorly; and it explores implications of this hypothesis. The paper also asks questions about the adequacy of systems of performance measurement of ambulance services in UK countries.

## 1. Introduction

Kelman (2006), in his review of Ferlie *et al.* (2005), was critical of its neglect of the uncomfortable fact that ‘Government does not perform as well as it should’. This uncomfortable fact is a product of unfavourable comparisons, in terms of dynamism and responsiveness, of services operating within government and normal markets (subject to well-known conditions) (Wolf, 1993). Indeed this contrast by Schulze (1977), pages 70–71, was cited by Enthoven (1985), page 10, in his argument for the introduction of an internal market for the National Health Service (NHS), to enable it to escape from its gridlocked form of the 1980s. As Wolf (1993) has argued, however, the reasons for the predominant and ineluctable source for failures of government services lie precisely in those circumstances that provide the rationale for these services being outside normal markets. Schulze (1977) highlighted that exit by failing providers was an intrinsic characteristic of the dynamism of normal markets. One reason why the Thatcher government's attempt to create appropriate incentives in the NHS, through the creation of an internal market, foundered was that Ministers could not allow exit by failing providers, as Ministers were responsible for ensuring good access to local services (Enthoven, 1999; Tuohy, 1999; Secretary of State for Health, 2000; Bevan and Robinson, 2005). So the ‘internal market’ evolved into one like the caucus race in *Alice in Wonderland*, in which ‘everyone must have prizes’ (Royce (1995) and Carroll (1975), page 49). Such difficulties illustrate why Kelman (2006) emphasized that a primary research concern of those studying public services ought to be tackling their underperformance. That concern is the subject of this paper: how can governments use performance measurement to put pressures on providers of public services that are analogous to those that normal markets put on private providers?

As Le Grand (2003), page 5, pointed out, in the post-war British welfare state, until the election of the Thatcher government in 1979, there was minimal interest in the design of external systems to improve the performance of providers of public services. This was because the guiding principle was the Panglossian assumption that all the key players were ‘knights’:

‘professionals … were concerned only with the interests of the people they were serving … politicians, civil servants, state bureaucrats, and managers were supposed accurately to divine social and individual needs in the areas concerned, to be motivated to meet those needs and hence operate services that did the best possible job from the resources available’.

This assumption explains why the shocking series of scandals in the NHS that came to light in England in 1998 went on for far too long (Smith, 1998; Abbasi, 1998; Bevan, 2008b) and is the only justification for the conventional British response to failure to deliver, namely rewarding failing organizations with extra resources. Following the Labour Government's policy of devolution (Greer, 2004), different systems of using performance measurement developed from 1998 within the countries of the UK. The Panglossian assumption that all key players in the NHS were knights continued as a guiding principle for the governments in Wales and Scotland; the government in England, however, sought, in 2000, to change the culture of the NHS from one that ‘bails out failure to one where it rewards success’ (Secretary of State for Health (2000), page 28), through the radical and controversial system of performance measurement by annual ‘star ratings’ of NHS organizations (known as ‘trusts’). Whereas the ‘internal market’ was designed to put financial pressure on failing providers in a way that threatened services for local populations, the star rating system was designed directly to put pressure only on those running failing providers to improve performance for local populations.

Star ratings were first applied in 2001 to acute trusts (Secretary of State for Health, 2001) and then extended to cover ambulance trusts in 2002 (Secretary of State for Health, 2002), and all types of trusts from 2003 until 2005, the final year of star ratings (Commission for Health Improvement, 2003a, b; Healthcare Commission, 2004, 2005a). This process gave each trust a score from zero to three stars based on performance against a small number of ‘key targets’ and a larger set of targets and indicators in a ‘balanced scorecard’. Trusts that failed against key targets, and were ‘zero rated’, were ‘named and shamed’ as ‘failing’, and their chief executives were at risk of losing their jobs. Trusts that performed well on both the key targets and the balanced scorecard, and achieved the highest rating of three stars, were rewarded by being publicly celebrated for being ‘high performing’ and granted ‘earned autonomy’ (Bevan and Hood, 2006a, b).

This paper examines the effects of the various systems of performance measurement in the countries of the UK for a common target for ambulance trusts, that 75% of emergency calls (made by someone telephoning 999), that may be immediately life threatening (category A), be met within 8 minutes, which we describe as the category A 8-minute target. This target was to be achieved in 2001 in England and Wales, and 4 and 6 years later in Northern Ireland and Scotland. The second main section of this paper gives contextual background by outlining a typology of systems of performance measurement, proposing a hypothesis of four requisite characteristics for a system to have had an effect and testing this through comparisons of different systems of hospital performance measurement, and outlining criticisms of the star rating system. The third main section of this paper describes the effects of various systems of performance measurement of ambulance response times in the UK. It outlines the development of targets for response times, describes the effects of star ratings on reported performance against these targets by different trusts in England and compares reported performance against the category A 8-minute target by different UK countries. That section also considers the basis and adequacy of target selection, definition and measurement, evidence of gaming in England (including detailed examination from analysis by one of us (RH) following concerns that were raised in the course of clinical governance reviews by the Commission for Health Improvement (CHI) of ambulance trusts). The paper concludes by raising disturbing questions for governments in the various UK countries from our study of targets for ambulance response times, considering recommendations on the development of, and research into, systems of performance measurement by a working party of the Royal Statistical Society in response to the Blair government's emphasis on these systems (Bird *et al.*, 2005).

## 2. Types and effects of different systems of performance measurement

### 2.1. A typology of performance measurement

Hood (2007) has described three types of systems of performance measurement: as general intelligence; in relation to targets; with measures being aggregated so that organizations can be ranked. This section considers the development of each type and its application to health care.

Intelligence systems have a long history in health care, going back to the publication of Florence Nightingale's analyses of hospital mortality rates (Nightingale, 1863). Since the 1990s, following the technological advances in computing and Web sites, there has been an explosion in the development of intelligence systems publishing clinical outcome indicators, but without resolving the problems that were identified in Florence Nightingale's analyses. Spiegelhalter (1999) pointed out that she clearly foresaw the three major problems that were cited in a survey of the publication of surgical mortality rates that were identified by Schneider and Epstein (1996) about 130 years later:

‘the inadequate control for the type of patient, data manipulation and the use of a single outcome measure such as mortality’.

Iezzoni (1996) pointed out that issues raised in the debate following publication of Nightingale's hospital mortality rates echo those which are cited frequently about contemporary efforts at measuring provider performance. These included problems of data quality, measurement (risk adjustment and the need to examine mortality 30 days after admission rather than in-hospital deaths), gaming (providers avoiding high risk patients because of fears of public exposure) and the public's ability to understand this information. These unresolved problems mean that there was, in the 1990s, intense and polarized debate about the benefits of publication of information on hospital performance, with different camps describing this as essential, desirable, inevitable and potentially dangerous (Marshall, Shekelle, Brook and Leatherman, 2000).

Although the use of targets also has a long history (Barber, 2007; Hood, 2007), Hood identified New Zealand as pioneering the comprehensive introduction of a system of target setting across government in the 1980s. This offered a model for the Blair government which, following its election in 1997, introduced the systematic setting of public service agreement targets, as part of an implicit contract between Her Majesty's Treasury and spending departments for their budgets for public services (James, 2004). (It was during this period that both England and Wales introduced the category A 8-minute target for ambulance trusts.)

The Thatcher government in the 1990s (before devolution) introduced the ranking system of league tables of performance of schools in terms of examination results, across the countries of the UK (West and Pennell, 2000; Department for Education and Skills, 2004). Hood (2007) has argued that what was distinctive and novel in the Blairite approach to performance measurement of public services was the development of government-mandated ranking systems. The differences between approaches to performance measurement in the UK are illustrated by the decisions, following devolution, of the government in England to maintain the publication of school league tables, and the governments in Wales and Scotland to abandon their publication (Hood, 2007). Bird *et al.* (2005) observed that school league tables are published in some states in the USA (California and Texas), but there has been legislation against their publication in New South Wales in Australia and the Republic of Ireland. For the NHS in each country, the government in England introduced the ranking system of star ratings, but the governments in Wales and Scotland, in their developments of performance measurement, deliberately eschewed these being published as ranking systems.

### 2.2. Comparisons of systems of hospital performance measurement

Using resources for performance measurement, rather than delivery, of health care can only be justified if the former has an influence on the latter: there is little justification on grounds of transparency alone if this has no effect. Spiegelhalter (1999) highlighted criticism by Codman (1917) of the ritual publication of hospital reports that gave details of morbidity tables and lists of operations which were intended to ‘impress the organisations and subscribers’ but were ‘not used by anybody’. The first systematic review of evaluations of systems of performance measurement, by Marshall, Shekelle, Brook and Leatherman (2000) and Marshall, Shekelle, Leatherman and Brook (2000) commented on the contrast between the scale of this activity and the lack of rigorous evaluation of its effects. A recent systematic review (Fung *et al.*, 2008) made the same point, emphasizing that the studies they had identified still focused on the same seven systems that had been examined by Marshall, Shekelle, Brook and Leatherman (2000); in particular on the cardiac surgery reporting system (CSRS) of New York State Department of Health. These systematic reviews produced evidence that enables us to examine three pathways through which performance measurement might result in improved performance. The first two of these, the change and selection pathways, were proposed by Berwick *et al.* (2003) and used by Fung *et al.* (2008). The change pathway assumes that providers are knights: that simply identifying scope for improvement leads to action, without there being any need for any incentive other than the provider's innate altruism and professionalism; thus, there is no need to make the results of the information available beyond the provider themselves. As Hibbard (2008) observed, the evidence suggests that this is a relatively weak stimulus to action. This finding was anticipated by Florence Nightingale in the 1850s in seeking to convey the urgent need to the government to improve the living conditions of army barracks in peacetime: her statistical analysis showed that these conditions were so appalling that the outcome was that, on the basis of comparisons of mortality rates with the civilian population outside,

‘1,500 soldiers good soldiers are as certainly killed by these neglects yearly as if they were drawn up on Salisbury plain and shot’.

She continually reminded herself that ‘reports are not self executive’ (Woodham-Smith (1970), pages 229–230). The selection pathway assumes that providers respond to the threat of patients as consumers using information in selecting providers, but, systematic reviews by Marshall, Shekelle, Brook and Leatherman (2000) and Marshall, Shekelle, Leatherman and Brook (2000) and Fung *et al.* (2008) found that patients did not respond as consumers in this way. In presenting the findings from that latest systematic review, at a seminar in January 2008, at the Health Foundation in London, Paul Shekelle observed that many of these studies were in the USA and showed that patients there did not use this information as consumers and, if that response has not materialized in the USA, with its emphasis on markets, then it is highly unlikely to be observed in other countries.

The systematic review of the evidence of effects of performance measurement systems by Fung *et al.* (2008) suggests that neither of the two pathways that were proposed by Berwick *et al.* (2003) for these systems to have an influence is effective. Hibbard (2008) has argued, however, that a third pathway of designing performance measurement that is directed at reputations can be a powerful driver of improvement. She has led research for over a decade into the requisite characteristics for a system of performance measurement to have an effect (see, for example, Hibbard *et al.* (1996, 1997, 2001, 2002, 2003, 2005a, b, 2007), Hibbard and Jewett (1997), Hibbard and Pawlson (2004) and Peters *et al.* (2007)). Hibbard *et al.* (2002) showed, in a controlled laboratory study, that comparative performance data were more likely to be used, if they were presented in a ranking system that made it easy to discern the high and low performers. Hibbard *et al.* (2003) proposed the hypothesis that, for a system of performance measurement to have an effect, it needs to satisfy four requisite characteristics: it must be

- a ranking system,
- published and widely disseminated,
- easily understood by the public (so that they can see which providers are performing well and poorly) and
- followed up by future reports (that show whether performance has improved or not).

Hibbard *et al.* (2003, 2005b) tested this hypothesis in a controlled experiment, based on a report, which ranked performance of 24 hospitals, in south central Wisconsin, in terms of quality of care. This report used two summary indices of adverse events (deaths and complications): within broad categories of surgery and non-surgery; across three areas of care (cardiac, maternity, and hip and knee). The report showed material variation (unlike insignificant differences in ranking in league tables) and highlighted hospitals with poor scores in maternity (eight) and cardiac care (three). The effects of reporting were assessed across three sets of hospitals: public report, private report and no report. For the public report set, a concerted effort was made to disseminate the report widely to the public: the report was available on a Web site; copies were inserted into the local newspaper, distributed by community groups and at libraries; the report attracted press coverage and generated substantial public interest. For the private report set, the report was supplied to managers only; the no-report set was not supplied with the report. This research design enables comparisons of the effects of the three pathways. If the change pathway were powerful, then there ought to be no difference between the public report and private report hospitals, but the public report set made significantly greater efforts to improve quality than the other two sets (Hibbard *et al.*, 2003, 2005b). The managers of hospitals in the public report set hospitals discounted the importance of the selection pathway: they did not see the report as affecting their market share (Hibbard *et al.*, 2003). Later analysis showed that these managers were correct:

‘There were no significant changes in market share among the hospitals in the public report from the pre to the post period … no shifts away from low-rated hospitals and no shifts toward higher-rated hospitals in overall discharges or in obstetric or cardiac care cases during any of the examined post-report time periods’

(Hibbard *et al.*, 2005b). The reputation pathway, however, was crucial: the managers of hospitals that had been shown to have been performing poorly in the public report group took action, because of their concerns over the effects of the report on their hospitals’ reputations. We now undertake two further tests of the hypothesis that, for a system of performance measurement to have an effect, this needs to be via the reputation pathway, through two comparisons of two hospital performance measurement systems, with reference to Hibbard's four requisite characteristics.

The first comparison is between two systems of reporting clinical outcome indicators. One is the much-studied CSRS of New York State Department of Health, which began in 1989 as the first statewide programme to produce public data on risk-adjusted death rates following coronary artery bypass graft surgery, and is the longest-running programme in the USA of this kind (Chassin, 2002). The other is the annual reports from the Clinical Resource and Audit Group (CRAG) in Scotland, which when these began in 1984 were at the forefront in Europe of public disclosure of such information (Mannion and Goddard, 2001; Clinical Resources and Audit Group, 2002).

The CSRS produces annual reports of observed, expected and risk-adjusted in-hospital 30-day mortality rates, by hospital and surgeon. Green and Wintfield (1995) observed

‘CSRS became the first profiling system with sufficient clinical detail to generate credible comparisons of providers’ outcomes. For this reason, CSRS has been recognized by many states and purchasers of care as the gold standard among systems of its kind.’

The CSRS satisfied three of the above four requisite characteristics: these annual reports are published and widely disseminated, although performance is not ranked, statistical outliers are identified (New York State Department of Health, 2006). The CSRS was used by hospitals and had an influence. There is controversy over the benefits from the dramatic improvements in reported performance: Chassin (2002) observed that

‘By 1992 New York had the lowest risk-adjusted mortality rate of any state in the nation and the most rapid rate of decline of any state with below-average mortality’;

Dranove *et al.* (2003) found, however, that such

‘mandatory reporting mechanisms inevitably give providers the incentive to decline to treat more difficult and complicated patients’.

What is of particular interest here is that, in the account by Chassin (2002) of how four hospitals went about the tasks of improvement, he emphasized that the selection and change pathways had no effect. The key driver of change was the reputation pathway through adverse publicity from the CSRS identifying outlier hospitals performing poorly (Chassin, 2002):

‘Market forces played no role. Managed care companies did not use the data in any way to reward better performing hospitals or to drive patients toward them. Nor did patients avoid high-mortality hospitals or seek out those with low mortality … the impetus to use the data to improve has been limited almost entirely to hospitals that have been named as outliers with poor performance … hospitals not faced with the opprobrium attached to being named as poorly performing outliers have largely failed to use the rich performance data to find ways to lift themselves from mediocrity to excellence.’

The CRAG's reports aimed to provide a benchmarking service for clinical staff by publishing comparative clinical outcome indicators across Scotland. The final report for 2002 (Clinical Resource and Audit Group, 2002) included two kinds of hospital clinical indicators (that used the only data the NHS collected routinely on outcomes following discharge from hospital): emergency readmission rates (for medical and surgical patients); mortality (or survival) after hospital treatment (for hip fracture, acute myocardial infarction, stroke and selected elective surgery). The CRAG reports essentially assumed a change pathway as the means through which the information that they produced would be used. These reports, which began before, and continued after, the internal market was introduced, were explicitly designed *not* to damage hospitals’ reputations: the last CRAG report (Clinical Resource and Audit Group (2002), page 2) emphasized that its information did not ‘constitute a ‘‘league table’’ of performance’. The CRAG reports were evaluated by a CRAG-funded Clinical Indicators Support Team (Clinical Resource and Audit Group (2002), pages 223–229) and Mannion and Goddard (2001, 2003). Despite the enormous effort that went into the production of these statistics, these evaluations found that they lacked credibility, because of the familiar problems of poor quality of data and inadequate adjustment for variation in casemix. These evaluations also found that the reports were difficult to interpret, lacked publicity and were not widely disseminated. Hence these reports did not satisfy Hibbard's four requisite characteristics. The two evaluations found that they had little influence: Mannion and Goddard (2003) found that these data were rarely used by staff in hospitals and the boards to which the hospitals were accountable, and general practitioners in discussions with patients.

The second comparison is a natural experiment between a ranking system, the star rating system in England, which was dominated by performance against targets for waiting times, and target systems for waiting times in Wales and Scotland, neither of which were part of ranking systems.

The star rating system in England satisfied Hibbard's four requisite characteristics and was designed to inflict reputational damage on hospitals performing poorly. Ranking through annual star rating was easy to understand, and the results were widely disseminated: they were published in the national and local newspapers and on Web sites, and featured in national and local television. Mannion *et al.* (2005a) emphasized that the star rating system stood out from most other systems of performance measurement in that hospital staff seemed to be highly engaged with information that was used in star ratings. They attributed this to ‘the effectiveness of the communication and dissemination strategy’ and ‘the comprehensibility and appeal of such a stark and simple way of presenting the data’. Star ratings obviously mattered for chief executives, as being zero rated resulted in damage to their reputations and threats to their jobs. In the first year (2001), the 12 zero-rated hospitals were described by the then Secretary of State for Health as the ‘dirty dozen’; six of their chief executives lost their jobs (Department of Health, 2002a). In the fourth year, the chief executives of the nine acute hospitals that were zero rated, were ‘named and shamed’ by the *Sun* (on October 21st, 2004), the newspaper with a circulation of over 3 million in Britain: a two-page spread had the heading ‘You make us sick! Scandal of Bosses running Britain's worst hospitals’ and claimed that they were delivering ‘squalid wards, long waiting times for treatment and rock-bottom staff morale’; a leader claimed that if they had been working in the private sector they would have ‘been sacked long ago’ (Whitfield, 2004). Mannion *et al.* (2005a) highlighted the pervasive nature of the damage to reputations that is caused by poor scores in star ratings on hospital staff. For one hospital, the effect of having been zero rated was described as having been ‘devastating’, ‘hit right down to the workforce—whereas bad reports usually hit senior management upwards’, and resulted in

‘Nurses demanding changing rooms because they didn't want to go outside [in uniform] because they were being accosted in the streets’.

Those from a one-star hospital described this as making people ‘who are currently employed here feel that they are working for a third class organisation’. More generally, star ratings were reported to affect recruitment of staff:

‘a high performance rating was ‘‘attractive’’ in that it signalled to potential recruits the impression that the trust was a ‘‘good’’ organisation to work for. In contrast, ‘‘low’’ performing trusts reported that a poor star rating contributed to their problems as many health professionals would be reluctant to join an organisation that had been publicly classified as under-performing.’

In Wales and Scotland, the target systems for long waiting times relied on the change pathway: that hospitals would know how their performance compared with targets set by government, and this alone would be enough to drive improvement. In each country there was neither systematic reporting to the public that ranked hospitals’ performance in a form analogous to star ratings, nor clarity in published information on waiting times: in Wales, breaches to targets were tolerated but not publicized (Auditor General for Wales (2005a), page 36); in Scotland, large numbers of patients actually waiting for treatment were excluded from published statistics (Auditor General for Scotland, 2001; Propper *et al.*, 2008). Each government's system of performance measurement lacked clarity in the priority of the various targets. In Wales, there was confusion over the relative priority of the various targets in the Service and Financial Framework and the government's targets for waiting times ‘not always [having] been clearly and consistently articulated or subject to clear and specific timescales’ (Auditor General for Wales (2005a), pages 36 and 41). In Scotland, the performance assessment framework was criticized for being ‘overly complex and inaccessible’ for the public and those working in the NHS (Farrar *et al.* (2004), pages 17–18). Both governments continued to reward failure. In Wales there were ‘neither strong incentives nor sanctions to improve waiting time performance’, and the perception was that

‘the current waiting time performance management regime effectively ‘‘rewarded failure’’ to deliver waiting time targets’

(Auditor General for Wales (2005a), pages 42 and 40). In Scotland, there was the perception of

‘perverse incentives … where ‘‘failing’’ Boards are ‘‘bailed out’’ with extra cash and those managing their finances well are not incentivised’

(Farrar *et al.* (2004), pages 20–21 and 4).

The natural experiment between star ratings in England, that satisfied the above four requisite characteristics, and the target systems in Wales and Scotland, that did not, has been subject to several studies to examine their effects on performance in waiting times, both over time and across countries at the national level: for England, Scotland and Wales (Bevan and Hood, 2006b; Auditor General for Wales, 2005a); for England and Wales (Bevan, 2008a); a detailed econometric analysis, for England and Scotland (Propper *et al.*, 2008). These comparisons have shown dramatic improvements in performance in England; initial deterioration in Wales and Scotland, and performance in England continuing to outstrip that of the other countries. Another cross-national comparison, by Willcox *et al.* (2007), of different countries’ attempts to tackle the problem of waiting times, compared Australia, Canada, England, New Zealand and Wales over the 6-year period 2000–2005. They summarized their finding as

‘Of the five countries, England has achieved the most sustained improvement, linked to major funding boosts, ambitious waiting-time targets, and a rigorous performance management system’

(Willcox *et al.*, 2007). There is a question of the extent to which the effects of the English system were due not only to their capacity to inflict reputational damage but also to the threats to the jobs of chief executives. This requires further research.

These various comparisons suggest that hospital performance measurement systems that satisfy Judith Hibbard's four requisite characteristics, in terms of their capacity to inflict damage on the reputations of hospitals performing poorly, had significant effects, whereas those systems that lacked that capacity had little or no effect. The importance of reputational damage as a key driver of change was also identified by Mannion and Davies (2002) in their interviews with experts in the USA in reporting health care performance: where reports of performance did have an effect, the underlying incentives were perceived to be, not financial, but ‘softer issues such as reputation, status and professional pride’. Hence Mannion and Davies highlighted the power of systems of ‘naming and shaming’ and that public reporting mattered, whether or not this was used by consumers, because ‘it makes providers pay more attention because they don't want to look bad’. Mannion and Goddard (2003) also emphasized that the US evidence is that public reporting has an impact ‘particularly where the organization is identified as performing poorly’. Florence Nightingale well understood reputational damage as a means of putting pressure on government to take action: she coined the battle-cry of those seeking reforms in the sanitary conditions of the peacetime army: ‘Our soldiers enlist to death in the barracks’ (Woodham-Smith (1970), page 229).

### 2.3. Criticisms of star ratings

The star rating system has been examined by the House of Commons Public Administration Select Committee (2003) and as part of the examination of systems of performance measurement by a working party of the Royal Statistical Society (Bird *et al.*, 2005). National auditors have examined responses to targets for hospital waiting times in England (National Audit Office, 2001a, b, 2004; Audit Commission, 2003), Wales (Auditor General for Wales, 2005a, b) and Scotland (Auditor General for Scotland, 2001, 2006a); responses to targets for ambulance response times in Scotland (National Audit Office, 1999; Auditor General for Scotland, 2006b), Wales (Auditor General for Wales, 2006) and England (Audit Commission, 2007). The CHI has published a detailed commentary on its star rating of the NHS in 2003 (Commission for Health Improvement, 2004a); it also reported on concerns over the way that trusts responded to targets for hospital waiting times (Commission for Health Improvement, 2004b) and ambulance response times (Commission for Health Improvement, 2003c), which were identified in the course of inspections of the implementation of the systems and processes of clinical governance in each NHS organization. There is already a substantial scholarly literature on the star rating system: Alvarez-Rosete *et al.* (2005), Bevan (2006, 2008a), Bevan and Cornwell (2006), Bevan and Hood (2004, 2006a, b), Brown and Lilford (2006), Burgess *et al.* (2003), De Bruijn (2007), Friedman and Kelman (2007), Hauck and Street (2007), Heath and Radcliffe (2007), Hood (2006, 2007), Jacobs *et al.* (2006), Jacobs and Goddard (2007), Jacobs and Smith (2004), Kelman and Friedman (2007), Klein (2002), Mannion *et al.* (2005a, b, c, Mannion and Goddard (2002), Marshall *et al.* (2003), Mays (2006), Patel *et al.* (2008), Propper *et al.* (2008), Rowan *et al.* (2004), Smith (2002, 2005), Snelling (2003), Spiegelhalter (2005a), Stevens (2004), Stevens *et al.* (2006), Sutherland and Leatherman (2006) and Willcox *et al.* (2007). Some of this literature has shown reported performance improving in England against the most important targets. All the criticisms of star ratings recognize its undeniable effect, but have also identified six significant general problems: in measuring what matters, selection of targets, nature of measures, aggregation for ranking, gaming and damaging morale (Table 1). The first four of these are essentially statistical; the last two raise questions about the behavioural influence of star ratings, which we see as consequences of any system that satisfies Judith Hibbard's four requisite characteristics, which is designed to inflict reputational damage on organizations performing poorly.

**Table 1:** Six problems with star ratings

*Type*	*Description*
Measuring what matters	Often the most important aspects of performance cannot be measured, and hence what is measured becomes important. School league tables based on examination results are an exemplar as a proxy measure of teacher performance, because of the difficulty of measuring the benefits of education from teaching. The general problem, of creating incentives for agents to respond to targets that omit key dimensions of performance, was modelled by Holmstrom and Milgrom (1991), who showed that neither using a limited set of good measures nor a larger set of poor measures would produce results that are free from significant distortion by gaming.
Selection	There are problems in assessing performance of complex organizations in selecting what ought to be included (and hence excluded) from the set of targets. For primary care organizations in England, for example, it is difficult to see a good rationale for the set of about 50 targets and indicators used in star ratings to cover their complex set of responsibilities (for providing primary and community health services, improving public health and commissioning secondary care) (Bevan, 2006).
Nature of measures	Performance indicators are often ‘tin openers’ rather than ‘dials’: ‘they do not give answers but prompt investigation and inquiry, and by themselves provide an incomplete and inaccurate picture’ (Carter *et al.*, 1995).
Aggregation for ranking	There are problems with methods of producing aggregate measures of performance for ranking systems (Jacobs and Goddard, 2007). League tables have been shown to be statistically unsound (Goldstein and Spiegelhalter, 1996; Marshall and Spiegelhalter, 1998). One criterion in the design of the star rating system was to avoid volatility from statistical noise in which there were substantial variations in performance from one year to the next. But the methods of determining whether an organization was three or two star were arcane (Klein, 2002), so complex that it was difficult for an organization to understand why its ranking had changed (Spiegelhalter, 2005a).
Gaming	When targets have been backed by high powered incentives in response to success and failure, a common effect has been gaming, both in centrally planned economies (Nove, 1958; Berliner, 1988; Kornai, 1994) and in the public sector (Smith, 1995).
Morale	Publicly reporting that hospitals were ‘failing’ (‘zero rated’ or one star) damaged the morale of their staff (Horton, 2004; Mannion *et al.*, 2005a, b).

If tackling failure were to have been ruled out on the grounds of damaging morale, then presumably the notorious scandals that beset the NHS in the late 1990s would have been allowed to continue for even longer than they did (Bevan, 2008b). Any system that satisfies Hibbard's four requisite characteristics will damage morale of those identified as performing poorly. Indeed it can be argued that damaging morale is necessary in the short term for creating the different atmosphere that is required to achieve improvement in the long term. Because star ratings were taken so seriously, they did encourage gaming, which was also a concern with the CSRS in New York. There are two corollaries of this. First, if we were only to adopt systems of performance measurement in which there were no incentive to game, these would be unlikely to be taken seriously, and hence fail to provide the external discipline to drive improvement of government services. Second, if we do take systems of performance measurement seriously, and design these to have an effect, then developing systems to counter gaming ought to be integral to the design of such systems.

The next section of this paper examines the effects of targets for ambulance response times to emergency calls in the various UK countries. There are two reasons why this is particularly interesting. First, we have a natural experiment between different approaches to using performance measurement: the category A 8-minute target was common to each UK country; but only in England was this subject to a system of performance measurement with Hibbard's requisite characteristics to effect change through the reputation pathway. Approaches in the other UK countries relied on the change pathway: that ambulance trusts would know how their performance compared with the target and this alone would be enough to drive improvement. Second, it seems so much easier to resolve the four statistical problems of star ratings that were identified above for ambulance services than, for example, for organizations as complex as acute care hospitals. A target for rapid responses by ambulances to life threatening emergency calls looks to be a good measure of what matters, as it reflects what we would expect ought to be a principal goal of those services. Indeed, the rationale for the selection of the category A 8-minute target was that

‘Clinical evidence shows that achievement of the target could save as many as 1,800 lives each year in people under 75 years suffering acute heart attacks’

(Healthcare Commission, 2005b). Furthermore, performance against this target requires only data that ought to be collected routinely and appears to be easy to measure.

## 3. The effects of targets on ambulance response times in the various UK countries

### 3.1. The development of targets and performance reporting

The NHS reorganization of 1974, which transferred responsibility for running ambulance services from local authorities in England and Wales to the NHS, revealed variations in standards for response times to emergency calls that lacked any good rationale. National standards that were set in 1974 became common standards across the UK and essentially remained the same for the next 20 years (based on an operational research consultancy study and hence known as ORCON standards): 50% and 95% of emergency calls ought to be met within 7 and 14 minutes in metropolitan areas; within 8 and 20 minutes in other areas (Department of Health and Social Security, 1974).

In England, following a review (National Health Service Executive, 1996), ambulance trusts were required to introduce a system of prioritization of emergency calls into three categories: A (may be immediately life threatening), B (serious but not immediately life threatening) and C (neither immediately life threatening nor serious). For category A calls ambulance trusts were set an ‘interim target’ of responding to 75% within 8 minutes by March 2001; for category B and C calls, 95% were to be met within 14 minutes for ‘urban’ or 19 minutes for ‘rural’ trusts (Department of Health, 1999a). (Urban and rural trusts were defined by whether the population density was more or less than 2.5 people per acre (Department of Health, 2000).) Performance against the new targets was reported publicly for each service as call prioritization was introduced from 1998–1999, and also within the star rating system from 2002 to 2005. Although the summary statistic for performance across England against the category A 8-minute target is reported from 2001–2002 only (Information Centre, 2007), this can be derived from annual statistics reported for the preceding years (Department of Health, 1999b, 2000, 2001).

In Wales, in April 1998, the Welsh Ambulance Trust was established, taking responsibility for ambulance services across the whole country. From April 1999, the same target of meeting 75% of category A calls within 8 minutes was introduced for all areas in Wales. This was to be achieved by the end of 2001, ‘with further progress thereafter’ (National Assembly for Wales, 2001). (Although there are differences in definition between England and Wales in what constitutes an emergency call, the Auditor General for Wales (2006), page 35, analysed a sample of 471 000 emergency calls and found that

‘there would have been only 0.6 per cent more Category ‘A’ calls in Wales had it applied the same call categorisations as England’

and hence concluded that it was valid to compare performance in these countries.) Performance was publicly reported for the Welsh Ambulance Service as a whole annually from April 1999, and for three different services within Wales (Central and West, North and South East Wales) quarterly from April 2001 (Auditor General for Wales, 2006). In contrast with England, failure by the Welsh Ambulance Service to meet the category A 8-minute target resulted, not in public censure, but in the imposition of successively less demanding ‘milestone’ targets being set for the percentages of category A calls to be met within 8 minutes: from April 2004, the target was reduced to 65% (the threshold for a service in England to have been zero rated); from April 2005 to 60% (which remains as the target for 2008–2009, supplemented by targets for 70% to be met within 9 minutes and 75% within 10 minutes) (Auditor General for Wales (2006), page 28, and Welsh Assembly Government (2007a), page 34).

The governments in Northern Ireland and Scotland introduced the category A 8-minute target to be achieved from 2005 (Rooker, 2006) and by 2007–2008 (Scottish Ambulance Service, 2004). In neither country was this target given the prominence that it was in England. We have been unable to find performance being reported publicly by governments of either country against that target on the Web sites of the Northern Ireland Statistics and Research Agency and Information Services Division Scotland. Hence our principal sources for data on performance in Northern Ireland and Scotland (which we report below) are from other sources: for Northern Ireland, a written answer to a question in the Houses of Lords (Rooker, 2006); for Scotland, from a report on the Welsh Ambulance Service, which compared the performance of the service in Wales with services in England and Scotland (Auditor General for Wales (2006), page 37), and Audit Scotland (2007).

### 3.2. Performance against targets

In England, star ratings for ambulance trusts included three targets for response times to emergency calls from 2002 to 2005: two were key targets, and one was in the balanced scorecard. The two key targets were that 75% and 95% of category A calls be met within 8 minutes and 14 or 19 minutes for urban or rural trusts. For the first 4 years of star ratings, there was a target in the balanced scorecard for category B and C calls to be met within 14 or 19 minutes. For the last 6 months of the final year of ratings, this was replaced by a third key target for category B calls only to be met within 14 or 19 minutes. There was no target in star ratings for category B and C calls to be met within 8 minutes. Table 2 gives information on the thresholds for the three key targets for emergency calls in the last year of star ratings for these trusts to be deemed to be ‘underachieving’ and ‘significantly underachieving’ against key targets, which resulted in two and six penalty points; a service would have been zero rated with six or more penalty points, and have been one star with four penalty points (Healthcare Commission, 2005b). (These thresholds were broadly consistent over the 5 years of star rating for category A calls.) The main challenge from star rating was meeting the category A 8-minute target (the old standard had required 50% of all emergency calls to be met within 7 or 8 minutes; the new key target required meeting 75% of category A calls within 8 minutes). The implications of performance against targets in the balanced scorecard were unclear in advance, as, until the final year of star ratings, this depended on relative performance against other trusts.

**Table 2:** Ambulance targets and thresholds for 2004–2005†

*Measure*	*Type of target*	*Significantly underachieved (%)*	*Underachieved (%)*	*Achieved (%)*
75% category A calls met <8 min	Key	<65	65–74	>74
95% category A calls met <14 min (urban) and <19 min (rural)	Key	<90	90–94	>94
75% category B and C calls met <8 min	None	—‡	—‡	—‡
95% category B and C calls met <14 min (urban) and <19 min (rural)	Key	<80	80–92	>92

† Source: Healthcare Commission (2005c).

‡ Not applicable.

Table 3 shows how performance changed over time in England for the four standards from 2000–2001, when over 30 trusts had implemented call prioritization, to 2004–2005. Before the introduction of star rating in 2002, few trusts had achieved the category A 8-minute target (although all were supposed to have done so by the end of March 2001). The effects of star ratings on performance for the subsequent years were different depending on the importance of the target in determining that rating. For the two key targets for category A calls, there were dramatic improvements against the 8-minute target, and some improvements against the 14- or 19-minute target. For the target in the balanced scorecard, for the first 3 years, for category B and C calls, there was little improvement against the target for 14 or 19 minutes during this period. When this ceased to be a target, for the first 6 months of 2004–2005 there was a worsening in performance. For category B and C calls within 8 minutes, there was no target in star ratings and virtually no improvement. The clear message is that trusts improved reported performance to avoid being classed as significantly underachieving against key targets (for category A calls less than 65% within 8 minutes and 90% within 14 or 19 minutes).

**Table 3:** Organizations’ performance against standards for emergency calls, from 2000–2001 to 2004–2005†

*Standard*	*Results for the following periods:*									
	*2000–2001*		*2001–2002*		*2002–2003*		*2003–2004*		*2004–2005*‡	
	*Number meeting target*	*Range (%)*	*Number meeting target*	*Range (%)*	*Number meeting target*	*Range (%)*	*Number meeting target*	*Range (%)*	*Number meeting target*	*Range (%)*
75% category A calls met <8 min	3	42–87	13	57–88	17	67–86	22	56–87	26	68–88
95% category A calls met <14 min (urban) and <19 min (rural)	19	83–100	24	86–100	22	88–100	21	84–100	25	90–100
75% category B and C calls met <8 min	1	35–82	1	40–82	1	32–82	1	29–83	1	27–85
95% category B and C calls met <14 min (urban) and <19 min (rural)	13	80–100	18	79–100	13	78–100	11	75–100	7	70–100
95% category B calls met <14 min (urban) and <19 min (rural)	—§	—§	—§	—§	—§	—§	—§	—§	10	68–100

† Source: Department of Health (2002b, 2004) (for 2000–2004) and Health and Social Care Information Centre (2005) (for 2005). The table is based on 31 organizations for every year except 2000–2001 when there were 32. In each case in this table performance is counted as having met the target if more than 75.0% or 95.0% of calls were met.

‡ In 2004–2005, there was no target for 95% of category B and C calls. A new target for 95% of category B calls applied to the last 6 months of that year only. Performance is reported here is for category B and C calls from April to September and category B calls from October to March.

§ Not applicable.

**Fig. 1:**
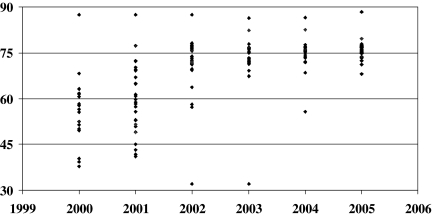
Percentage of category A calls met within 8 minutes (2000–2005) (source: Department of Health (2002b, 2004) (for 2000–2004) and Health and Social Care Information Centre (2005) (for 2005))

Fig. 1 gives performance for the years ending in March from 2000 to 2005 for each of 28 trusts (which were unaffected by mergers) and shows the transformation in reported performance in England for meeting the category A 8-minute target, before star rating (for the years ending in March 2000 and 2001), and after (for the years ending in March from 2002 to 2005). In the year before star ratings, only three trusts achieved the category A 8-minute target; 17 met less than 65% of such calls (which, if maintained, would have resulted in a zero rating) and, of those 17, five met less than 50% of such calls within 8 minutes (four of these were classed as rural, but the worst, with only a 42% response rate, was the London Ambulance Service, which had suffered from a catastrophic failure of a computer system in the early 1990s).

To achieve more demanding targets for response times to emergency calls when these vary and are uncertain, it is obviously necessary to manage supply better to meet peaks in demand. There is limited information on how this was achieved by ambulance services in England. In Essex new management implemented measures that improved staff morale and focused on changing supply to achieve the key targets. This included developing out-of-hours care, developing emergency care practitioners, improved staffing and buying new emergency vehicles and equipment (Bevington *et al.*, 2004). The Auditor General for Wales (2006), page 104, argued that

‘despite the record of poor performance and of failures in key areas of business management in the Welsh Ambulance Service, there were grounds for optimism’,

as other ambulance trusts in England had faced ‘somewhat similar situations but been able to turn themselves round, given time’. The Auditor General for Wales (2006), pages 105–106, gave two case-studies of such turnarounds in London and East Anglia Ambulance Trusts, which were attributed to

‘sound planning, change management and budgeting, … strong programme management, the need to refresh plans, and the need to consider the management capacity required to support and maintain change’.

The London Ambulance Service implemented a service improvement plan, which improved the percentage of category A response times met within 8 minutes from 40% to 75.1%.

**Fig. 2:**
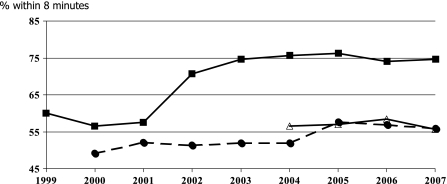
Percentage of category A calls met within 8 minutes, in England (▪), Wales (•) and Scotland (Δ) (sources: England, Department of Health (1999a, 2000, 2001) (for 1999–2001) and Information Centre (2007) (for 2002–2007); Wales, National Assembly for Wales (2005) (for 2000–2004), Auditor General for Wales (2006), page 37 (for 2005 and 2006), and Welsh Assembly Government (2007b) (for 2007); Scotland, Auditor General for Wales (2006), page 37, and Audit Scotland (2007), page 2 (for 2007))

Fig. 2 gives performance (where data are available) for England, Wales and Scotland, from 1999–2000 to 2005–2006. This shows that the service in England achieved the 75% target on average from 2003 (the reduction in 2006 is due to an adjustment to reflect concerns about data recording in six English trusts—this is discussed below). The service in Wales, which has achieved neither the 75% target set in 2001, nor the 65% target set in 2004 nor the 60% target set in 2005, was the subject of a damning report by the Auditor General for Wales (2006) (see below). Fig. 2 shows that, since 2004, the ambulance service in Scotland had a similar performance to that of Wales, meeting less than 60% of category A calls within 8 minutes. The only information that we have been able to find on the performance of the service in Northern Ireland was that, in 2005–2006, this met 51% of category A calls within 8 minutes (Rooker, 2006). These standards of performance seem to have had little resonance in either Scotland or Northern Ireland.

The failure to improve performance of the Welsh ambulance services was attributed by the Auditor General for Wales (2006) to ‘problems of strategy, leadership, governance, process, infrastructure and systems, people and culture’ (page 8). The reasons for the mismatch between supply of, and demand for, services included inflexible shift patterns and deployment, inadequate supply of ambulances (due to failures in procurement of new ambulances, ambulances being old with high failure rates and insufficient spare fleet capacity) and inadequate systems (due to failures in procurement of new systems, failure to invest in satellite navigation and problems with the radio network) (pages 14–15). There were, however, within Wales, significant variations in performance: the percentages of category A calls met within 8 minutes in 2005–2006 ranged from 70% to 40% (page 32). Some (but not all) of the poor performers served a sparsely populated area, but the report found little evidence of attempts by those services ‘to mitigate these problems through seeking to develop new models of service delivery’. One weakness of governance was the lack of benchmarking against other ambulance services (page 63). The report did this and found that the service in Wales had higher spend *per capita* than rural services in England but worse performance (pages 54 and 36).

### 3.3. Problems of target selection

Pell *et al.* (2001) showed that rapid responses to emergency calls following cardiac arrest (abrupt cessation of the pump function of the heart that without prompt intervention will lead to death) was the best way of reducing mortality from coronary heart disease in the UK, which has been, and despite recent improvements continues to be, relatively high. (Among developed countries, only Ireland and Finland have a higher rate than the UK (Allender *et al.*, 2006).) Pell *et al.* (2001) emphasized that most deaths from cardiac arrest occurred out of hospital: about 75% of all deaths and 91% of people under 55 years of age. Their analysis of data from Scotland of cases with cardiac arrests between 1991 and 1998, who had been seen within 14 minutes, found that only 6% survived to hospital discharge. They estimated that, if these cases had been reached in 8 or 5 minutes, that percentage would have increased to between 8% and 10%. This study gives a basis to the rationale for the selection of the category A 8-minute target: that

‘Clinical evidence shows that achievement of the target could save as many as 1,800 lives each year in people under 75 years suffering acute heart attacks’

(Healthcare Commission, 2005b). There are, however, two problems in making this connection.

The first is that the quicker the response the better, and Pell *et al.* (2001) emphasized advantages of equipping other first responders with ‘intelligent’ defibrillators to provide cardiopulmonary resuscitation in less than 8 minutes: such as firefighters (as the fire service has many more stations than the ambulance service and 90% of vehicles are required to attend the scene of a fire within 5 minutes), police or community volunteers. The second is the law that Charles Goodhart proposed following his analysis of the failure of the UK Government's reliance on money supply targets in the 1980s to control inflation: ‘Any observed statistical regularity will tend to collapse once pressure is placed on it for control purposes’ (Goodhart (1984), page 94). Goodhart's law means that, although statistical analysis of historical data may suggest a relationship between a target and an outcome, once the target is used, the people who generate the data for the target may change their behaviour so the relationship breaks down. As we explain below, gaming in response to the category A 8-minute target undermined the promised benefits of its realization. As Heath and Radcliffe (2007) argued ‘assuming that more lives will be saved if response targets are met is too simplistic’.

One way of taking account of the benefits of quick response times across different services and gaming would be to develop measures of performance, using Donabedian's classic framework for improving quality of care (Donabedian, 2005), in terms of structure, process and outcome. This suggests generating, in addition to the current targets of process, one of structure, the availability of defibrillators in ambulances, and one of outcomes, return of spontaneous circulation rates following cardiac arrest (as argued by Heath and Radcliffe (2007)). This would help to counter gaming in response to the current targets for process only: for example, if return of spontaneous circulation rates did not increase in line with increases in response times, this would raise questions about how the reported increase in response times had been achieved. This would also help to indicate the extent to which there was co-ordination across emergency services. Indeed one of the government's ambitions for performance measurement in England, through the system that became star ratings (then described as ‘traffic lights’), was to take account of the way in which different organizations worked in partnership with others in performing on key shared objectives across the local ‘health economy’ (Secretary of State for Health (2000), page 64). One of the weaknesses of the star rating system was the way that it failed to do this: by assessing different health services separately, the system encouraged one service to achieve its own targets by gaming even if this adversely affected another. (A notorious example was that some hospitals kept patients waiting in ambulances outside the hospital until the hospital could be confident that the patient could be seen in its accident and emergency department within the 4-hour target (Commission for Health Improvement, 2004b).)

### 3.4. Problems of target definition

The official definitions in timing ambulance responses were that the clock ought to start when details of the telephone call had been ascertained and stop when the emergency response vehicle, dispatched by, and accountable to, the ambulance service, arrived at the scene of the incident. (This could include a paramedic on a motorbike or in a car or an approved ‘first responder’ who was not employed by the ambulance service, such as a doctor, policeman or fireman (Department of Health, 1999b, 2000).) In practice, however, there were troubling variations in the recording of response times (Commission for Health Improvement, 2003c). The definition of what constituted a category A call was left to local discretion, which resulted in fivefold variation across trusts in the percentages of emergency calls that were classified as category A. This variation persisted from 2001 to 2005. Such extreme disparities must have meant that different trusts consistently made different judgements over what did, and did not, constitute a life threatening emergency. The Commission for Health Improvement (2003c) recommended that these problems of definition be tackled by detailed analysis of the various approaches to categorization. The ambiguity over definition of category A calls raises a fundamental question about the achievement of the 8-minute target.

### 3.5. Gaming in England

The problems that we have identified in definition and recording were raised in evidence in 2002 to the House of Commons Public Administration Select Committee (2002) and obviously presented opportunities for gaming. We discuss three sets of problems here, identified in the CHI's clinical governance reviews (Commission for Health Improvement, 2003c): consequences of the intense focus on the category A 8-minute target, definition of category A calls and manual ‘correction’ of response times.

The intense focus on the category A 8-minute target gave rise to three concerns. First, as urgent calls for an ambulance from general practitioners for patients were not classed as category A, they could be given lower priority. Second, a common view was that ‘to get there in 8.01 minutes and save the patient is seen as a failure’ (Commission for Health Improvement (2003c), page 9). Third, it was alleged that some trusts concentrated ambulances in densely populated areas (where the bulk of calls could be reached within 8 minutes) at the expense of patients in rural areas. This logical response to the category A 8-minute target is a vivid illustration of a trade-off between efficiency (meeting as many calls as possible within 8 minutes) and equity (that access depends on need and not where people live). But we doubt whether those who framed this policy would have regarded such relocations as an acceptable response. (Although a limit set was on how much worse performance could be for calls that took longer than 8 minutes, as another key target was that 95% of category A calls receive a response within 14 minutes or 19 minutes for urban or rural trusts.)

Staff at many ambulance trusts alleged that there had been exploitation of the ambiguity over the definitions of category A calls to game the system: by classifying incidents as category A if the control room believed that they could be met in 8 minutes, and category B and C if not, or through selectively reclassifying calls following the conclusion of the incident. The CHI, however, found hard evidence that this had occurred once only (which resulted in this practice being stopped (Commission for Health Improvement (2003c), page 15)).

**Fig. 3:**
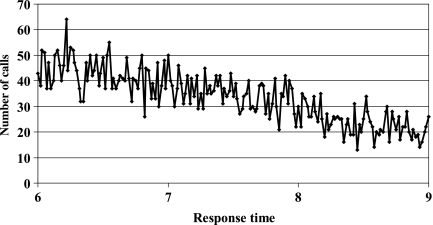
Example of a ‘noisy’ decline in response times to category A calls by one service (source: Commission for Health Improvement (2003c))

**Fig. 4:**
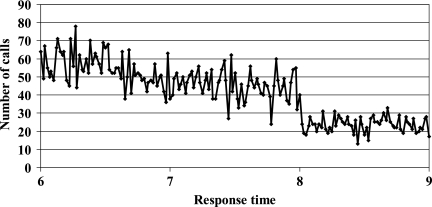
Marginal discontinuity in frequency of response times to category A calls by one service (source: Commission for Health Improvement (2003c))

**Fig. 5:**
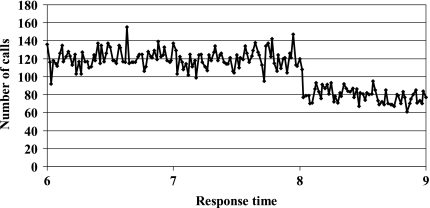
Slope in frequency of response times to category A calls around 8 minutes by one service (source: Commission for Health Improvement (2003c))

**Fig. 6:**
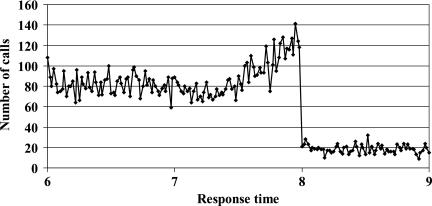
Bulge in frequency of response times to category A calls around 8 minutes by one service (source: Commission for Health Improvement (2003c))

**Fig. 7:**
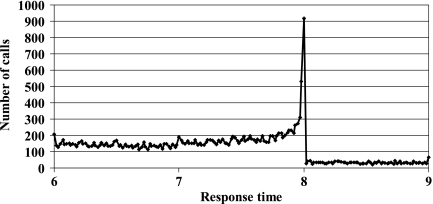
Spike in frequency of response times to category A calls at 8 minutes by one service (source: Commission for Health Improvement (2003c))

**Fig. 8:**
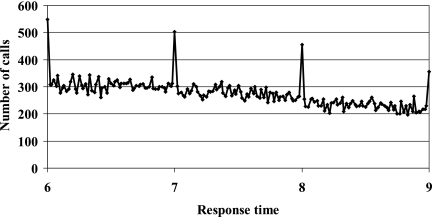
Spikes in frequency of response times to category A calls at each minute by one service (source: Commission for Health Improvement (2003c))

The CHI found out that, in one service, the times of responses taking longer than 8 minutes had been ‘corrected’ to be recorded as taking less than 8 minutes. This was mainly because targets created a culture in which staff felt under pressure to record the ‘right’ answer. A subsequent reanalysis of all English ambulance service data showed that such manual ‘corrections’ had been undertaken in around a third of trusts. This investigation concentrated on the frequency distributions of response times and identified oddly shaped frequency distributions that exhibited sharp discontinuities around the 8-minute target (Commission for Health Improvement, 2003c, d). Fig. 3 gives the expected (‘uncorrected’) distribution from one service: of a ‘noisy’ decline in numbers of responses over time with no obvious jump around the 8-minute target. Figs 4, 5, 6, 7 and 8 are examples from different trusts of different types of ‘corrections’: Fig. 4 shows a marginal discontinuity, which becomes more marked in Figs 5 and 6; Fig. 7 has a dramatic spike at 8 minutes. In each of these there is a clear implication that calls just over 8 minutes were reassigned to give a response time of 8 minutes or less. Fig. 8 shows a spike at each minute; in this case the problem was not one of gaming, but such poor data recording systems that for a significant proportion of calls manual entries were recorded to the nearest minute. The CHI estimated, however, that the effect of the most dramatic corrections would have improved performance by at most 6%; hence star ratings did produce substantial improvements in performance against the category A 8-minute target (of up to 20% since 1999).

For trusts where there was a clear discontinuity at precisely 8 minutes for category A calls, the CHI undertook statistical tests to determine the extent of the discontinuity by performing a weighted regression of the count of category A calls on the response times (weighted by the number of calls), for the data from 7:01 minutes to 8:00 minutes. Using the results from the regression analysis, the total number of predicted calls between 8:01 minutes and 9:00 minutes was compared with the actual number of calls recorded in the same time period. In the majority of cases where discontinuity was judged significant, the actual calls met between 8:01 minutes and 9:00 minutes fell outside the 95% prediction interval.

## 4. Discussion

In this discussion we begin by considering troubling questions for governments in the UK that were raised by our analysis of performance of ambulance services against targets for ambulance response times. We then consider recommendations on the development of, and research into, systems of performance measurement by the Royal Statistical Society (Bird *et al.*, 2005).

### 4.1. Ambulance response times in UK countries

The UK had, and continues to have, high mortality from coronary heart disease, which could be reduced by rapid responses to cardiac arrests by emergency services. This offers a rationale for the target that 75% of category A calls be met within 8 minutes for ambulance services, which has been proposed for each UK country. Only in England, however, has that target been met: on average, in each of the other countries in the UK, less than 60% of category A calls have been met within 8 minutes. So, if those services were to have been subjected to the English system of star rating, they would have been zero rated and publicly named as failing. At first sight it seems bizarre that devolution has allowed very different approaches to performance management, which have resulted in such marked differences in performance between the UK countries against a target, which is held in common, and relates to a vital emergency service, and where failure to meet it may mean that lives are being lost. One explanation was suggested by Hood (2007), page 97, who has argued that the larger scale of England creates a greater degree of relational distance between governments and providers of public services than in Scotland and Wales, where there are ‘smaller societies with more tightly-linked and overlapping political and social elites’. Bevan (2008a) suggested that this explains their reluctance to introduce systems of performance measurement that inflict reputational damage on poor performers. Alvarez-Rosete *et al.* (2005) showed that differences in performance of NHSs of the various UK countries were not caused by differences in aggregate levels of funding. So is variation in response times to the category A 8-minute target another example of the price of devolution being to condemn populations in Scotland, Wales and Northern Ireland to underperforming public services? Alvarez-Rosete *et al.* (2005) highlighted problems, following devolution, in finding consistent data across each country for the most basic comparisons. After 2003, for example, the Office for National Statistics stopped publishing information on hospital waiting times across the UK (Office for National Statistics, 2004). As we have made clear, we have found it difficult to compare performance of ambulance services across the UK. It seems to us essential that the Office for National Statistics (or some other body with similar authority and independence) is empowered to specify and collect basic comparative data on health care and to publish these so that people in each country are aware of the differences emerging through devolution. This would require each country to publish data that are comparable with those for England on performance of ambulance response times. Such requirements might inflict the necessary reputational damage on ambulance services and governments to encourage them to remedy serious underperformance.

Another set of questions is raised by variations in responses to, and the timing of, the setting of the category A 8-minute target. Why did continued failure of the service in Wales to achieve that target result in successively less demanding milestone targets being set? Why did it take so long for there to be a thorough investigation of that service found to have been wanting in all key elements of management? Why has a similarly dismal level of performance in Northern Ireland and Scotland not resulted in similar concerns? It may be argued that the answer to that question is that the category A 8-minute target was only set in those countries to be achieved later (2005 and 2007), whereas in England and Wales this was intended to be achieved from 2001. But that only prompts an even more troubling question: if it seemed a good idea to introduce this in England and Wales in 1996 to be achieved in 2001, as a way of ‘saving lives’, why did it make sense to delay requiring its achievement until 4 and 6 years later in Scotland and Northern Ireland? In particular, a report by the Comptroller and Auditor General (National Audit Office, 1999) made similar criticisms about performance of the ambulance service in Scotland to that made of the service in Wales: in terms of failure to meet the target that then applied in Scotland (in Glasgow only one in three ambulances responded to an emergency call, within 7 minutes, against a target of one in two), because supply did not match peaks in demand.

In England, until 2001, performance of ambulance services fell well short of the category A 8-minute target. After the star rating system applied to ambulance services from 2002, these trusts responded to produce improvements in their *reported* performance for the key targets of the star rating system, in particular the category A 8-minute target; but they did not improve their reported performance for a standard that was excluded from that system, namely meeting category B and C calls met within 8 minutes. There are, however, questions over the extent to which hitting the category A 8-minute target translates into commensurate improvements in outcomes, because of concerns over the selection of that target and its vulnerability to gaming. The striking evidence of gaming in the manual recording of response times appears to be less troubling than other concerns over definitions of what constituted a life threatening emergency (category A) call, and when the clock started and stopped for responses to these calls. These problems were raised in evidence in 2002 to the House of Commons Public Administration Select Committee (2002). Why was nothing done to address them within the star rating system, by, for example, developing, in addition to these measures of process in response times, measures of structure (availability of defibrillators) and outcome (return of spontaneous circulation)? We are delighted to see that the proposed new national target indicators for ambulance trusts in England that were proposed by the Healthcare Commission (2007) for 2007–2008 are directed at auditing outcomes including those for cardiac arrest.

In 2005, the Healthcare Commission published the final set of star ratings of ambulance trusts, and the Department of Health (2005) a report on ambulance services in England, following a strategic review. That report recommended that there be only two national response time requirements for category A calls only, that 75% be met within 8 minutes and 95% within 19 minutes; performance requirements for responding to patients whose general practitioner calls 999 on their behalf should be the same as for other 999 calls. The report asserted that only 10% of all emergency calls were for life threatening emergencies and changed the definition of the start of the clock to when the emergency call is connected to the ambulance control room. Why did the report, however, neither clarify ambiguity over definition of life threatening emergencies nor recommend scrutiny to counter gaming?

In 2006, after whistle-blowers at one trust contacted the NHS counter-fraud service, the Department of Health reported that an audit had found that six out of 31 trusts had failed accurately to record the actual response times to the most serious life threatening emergency call with a disturbingly familiar identification of well-known weaknesses (Carvel, 2006):

‘Some did not start the clock as soon as a 999 call was received. Others did not synchronise the clocks on the emergency switchboard with those used by paramedics. In some cases, ambulance organisations recategorised the urgency of the call after the job was done to make it fit the response time achieved rather than the priority given when the original call was made. This would have allowed staff to downgrade an emergency if the ambulance arrived late.’

In 2007, the Audit Commission (2007) issued a public interest report on the former Wiltshire Ambulance Service NHS Trust, following an audit. The report found, between April 2005 and July 2006, that control room staff had manually changed response times for nearly 600 category A and nearly 90 category B calls. The report (page 13) pointed out that

‘In a culture where managers’ jobs depend on achieving specific targets, there will be pressure to meet those targets. If a system is properly managed, it can be used to monitor and improve performance as a whole. But, a system can also be used to manipulate the data, rather than changing the way the service is delivered. Data that is easy to manipulate gives people both the motive and opportunity to do so.’

This behaviour was noted up to 3 years after the CHI report into precisely this type of manipulation had found the Wiltshire Ambulance Service to be one of the worst offenders, and after at least two changes in leadership. So the apparent success of ambulance trusts in England in meeting the category A 8-minute target is tarnished by the failure to design systems to tackle gaming identified as a problem in 2002. The failure to tackle gaming in response to targets from the outset was a general weakness of the star rating system (Bevan and Hood, 2006a) and of the Blairite regime of performance measurement (Hood, 2006). Why was so little done in England to tackle such persistent problems once these became so obvious for ambulance services: is this the most insidious form of gaming in which improvement in *reported* performance was seen as the prerequisite, regardless of what was actually happening? In the chapter in which Barber (2007), pages 264–287, reviewed the effect of the Prime Minister's Delivery Unit, he considered Riddell's observation about the Blair government having been given insufficient credit for firmly based achievements in improving public services (Riddell, 2005). Now it seems to us that a good reason for this was that the evidence of gaming, and failure to tackle this gaming, undermined the credibility of improvements in reported performance. To our surprise this is not even considered by Barber in that chapter.

### 4.2. General lessons from the case-study

The Royal Statistical Society's working party (Bird *et al.* (2005), page 4) sought to

‘present a practical perspective on performance measurement that can help to resolve critical issues in the design, analysis and reporting of performance indicators in the public services’.

Two of these concerned the need for consideration of ethical issues, and research into robust methods for evaluating new government policies. We summarize the other recommendations as follows, that systems of performance measurement need:

- to be evaluated to show that their benefits outweigh their burden;
- research into relative merits of different dissemination strategies for the public release of data;
- substantial input from individuals and/or institutions being monitored;
- a wide-ranging educational effort about the role and interpretation of data;
- to take account of variations and uncertainty;
- a detailed protocol, clearly specified objectives, clear definition of performance indicators and methodological rigour;
- to be designed so that counterproductive behaviour is discouraged and subject to independent scrutiny.

We strongly support the first recommendation. Developments in computing have resulted in a growth industry in performance measurement of health services. Little of this vast activity has been evaluated and, where it has, the typical finding is that, because they were designed for the change or selection pathways, they have had little or no effect. We would pose the first recommendation more urgently starkly and simply to emphasize the importance of finding out whether a system of performance measurement has *any* discernible effect. It seems to us that there has been a tendency in criticisms of the Blairite regime of performance measurement to overlook one of its most important characteristics, namely that this regime had an effect, in contrast with many others that seem to have provided only employment for those who produce statistics.

Hibbard *et al.* (2003) proposed the importance of a third pathway through which a system of performance measurement can have an influence, namely the reputation pathway. She has also identified four requisite characteristics of a system to have an effect in this way: that it be a ranking system, published and widely disseminated, easily understood by the public and followed up by future reports. This paper has summarized evidence to test that hypothesis from three comparisons of systems of hospital performance measurement. The systems that satisfied all or most of those requisite characteristics did have an effect, and those that did not failed to do so. These studies, and other evidence, suggest that the key driver in the systems that did have an effect were what Mannion and Davies (2002) identified as the ‘softer issues’: not finance, but ‘reputation, status and professional pride’. In particular the overriding concern was the damage to reputations from providers publicly ‘named and shamed’ as being the worst performers. This paper gives another test of that hypothesis of performance in a natural experiment: different countries of the UK have adopted different systems of performance measurement of ambulance services against the common category A 8-minute target. Only in England was there a system that satisfied Judith Hibbard's four requisite characteristics, and only there has this target been achieved. We see these requisite characteristics, and evidence from systems that do and do not satisfy them, as giving a new perspective on the other six recommendations.

The second recommendation concerns research. We see the need for more research directed at testing the hypothesis proposed by Hibbard *et al.* (2003), and the implication that, to have an effect, systems need to be designed to inflict reputational damage on those which are shown to be performing poorly. In particular, for the English system of star ratings, research is needed: to assess the importance in that system of reputational damage and threats to the jobs of chief executives of NHS trusts; of how organizations transformed their capacity to match supply to variable and uncertain demands in reducing hospital waiting times for admission and ambulance response times to emergency calls. The third recommendation (on substantial input from those being monitored) conceals a potential threat to effective systems of performance measurement: as Hibbard *et al.* (2003) observed

‘providers will vigorously oppose an approach that explicitly ranks or identifies top and bottom performers. However, it may be this very strategy that makes the difference between motivation to improve and no motivation.’

The fourth recommendation calls for ‘a wide-ranging educational effort about the role and interpretation of data’. We would instead recast the fifth and sixth recommendations, on statistical developments in performance measurement, as requiring those that are statistically sound but can be easily understood by the public, e.g. Spiegelhalter's development of funnel plots as a way of understanding variation (Spiegelhalter, 2005b). A particularly challenging problem is the development of sound systems of aggregation to produce a ranking of performance. Our study of targeting ambulance response times demonstrates the importance of the sixth and seventh recommendations: the need for clearly defined targets; detailed protocols for reporting of data on performance; assessment designed to discourage counterproductive behaviour; independent scrutiny of reported performance to achieve proper accountability and to provide a check on counterproductive behaviours. In conclusion we emphasize that what matters in designing systems that will have an effect is to make countering gaming integral to that design.
